# Prevalence of disability and associated factors in Dabat Health and Demographic Surveillance System site, northwest Ethiopia

**DOI:** 10.1186/s12889-017-4763-0

**Published:** 2017-10-02

**Authors:** Mulugeta Bayisa Chala, Solomon Mekonnen, Gashaw Andargie, Yigzaw Kebede, Mezgebu Yitayal, Kassahun Alemu, Tadesse Awoke, Mamo Wubeshet, Temesgen Azmeraw, Melkamu Birku, Amare Tariku, Abebaw Gebeyehu, Alemayehu Shimeka, Zemichael Gizaw

**Affiliations:** 1 Department of Physiotherapy, School of Medicine, College of Medicine and Health Science, University of Gondar, P.o. Box 196, Gondar, Ethiopia; 2 Department of Human Nutrition, Institute of Public Health, College of Medicine and Health Sciences, University of Gondar, Gondar, Ethiopia; 3 Department of Health Service Management and Health Economics, Institute of Public Health College of Medicine, University of Gondar, Gondar, Ethiopia; 4 Department of Epidemiology and Biostatistics, Institute of Public Health College of Medicine and Health Science, University of Gondar, Gondar, Ethiopia; 5 Department of Environmental and Occupational Health and Safety, Institute of Public Health College of Medicine and Health Science, University of Gondar, Gondar, Ethiopia; 6 Dabat Research Centre Health and Demographic Surveillance System, Institute of Public Health College of Medicine and Health Science, University of Gondar, Gondar, Ethiopia; 7 Department of Reproductive Health, Institute of Public Health, College of Medicine and Health Science, University of Gondar, Gondar, Ethiopia

**Keywords:** Disability, Vision disability, HDSS, Dabat, Ethiopia

## Abstract

**Background:**

Despite the high burden of disability in Ethiopia, little is known about it, particularly in the study area. Hence, this study aimed to investigate the prevalence and factors associated with disability at Dabat Health and Demographic Surveillance System (HDSS) site, northwest Ethiopia.

**Method:**

A population-based study was conducted from October to December 2014 at Dabat HDSS site. A total of 67,395 people were included in the study. The multivariable binary logistic regression analysis was employed to identify factors associated with disability. The Adjusted Odds Ratio (AOR) with a 95% Confidence Interval (CI) was estimated to show the strength of association. A *p*-value of <0.05 was used to declare statistical significance.

**Results:**

One thousand two hundred twenty-eight individuals were reported to have a disability giving a prevalence rate of 1.82%, of which, about 39% was related to a vision disability. The high odds of disability were observed among the elderly (≥50 years) [AOR: 4.49; 95% CI: 1.95, 10.33], severely food in-secured [AOR: 2.11; 95% CI: 1.59, 2.80], and separated marital status [AOR: 7.52; 95% CI: 1.18, 47.84]. While having a paid job [AOR: 0.46; 95% CI: 0.28, 0.77], being in the richest quintile [AOR: 0.55; 95% CI: 0.41, 0.75], and high engagement in work-related physical activities [AOR: 0.36; 95% CI: 0.27, 0.49] were inversely associated with the disability.

**Conclusion:**

Disability is a major public health problem, and the burden is noticeable in the study area. Vision disability is the highest of all disabilities. Thus, efforts must be made on educating the public about disability and injury prevention. Measures that reduce disability should target the elderly, the poorer and the unemployed segment of the population.

## Background

The World Health Organization estimates that globally around 1 billion people (15%) live with some sort of disability [1]; the majority live in resource-limited settings [2, 3]. This number is increasing due to the rise of an aging population, advancement of medical care, and population growth across the world [3]. However, the subject is considered as a human right and global health issues as well as an agenda for development [2].

Disability is defined as having difficulties with performing activities of daily living (ADL), and the phenomena are expressed as an interaction between an individual’s health condition and the environment he or she is living in [4, 5]. The Washington group defined disability as having at least a severe difficulty or limitation in performing key ADL, such as sight, hearing, walking or climbing steps, remembering, or concentrating [6].

People with disability face different challenges during their lifetime. This can be explained by social exclusion, stigma, severe health challenges, limited access to school and business [4]. In addition, it affects not only the person’s individual life but also his or her participation and role in society [2]. The difficulties and barriers experienced by people with disabilities are not only due to their own health conditions, but also because of inadequate policies and standards empowering and supporting these people. This is usually reflected in negative attitudes towards them, prejudices, and the inaccessibility of services [2]. In fact, disability is also linked with poverty [5, 7, 8], and people living with disabilities in developing countries face many challenges in their daily life.

Disability is caused by several factors, such as poor living conditions, poor nutrition, lack of health and sanitation facilities, different forms of accidents and injuries [7], congenital malformation, psychological dysfunctions, and birth related incidents [9].

In order to create equal opportunities in every sphere of their life, many countries, including Ethiopia, signed the convention of the rights of people with disabilities ratified by the United Nations in 2006 [2]. However, there is a major gap between implementing the stated convention and the day to day life of people with a disability. Besides, the convention urges the member countries to establish a proper mechanism that ensures a regular collection of data at the population level [10].

Ethiopia has also signed the African Decades of Disabled Persons (SADPD), which was established in South Africa in 2004 with the responsibility of coordinating efforts and resources on disability programs in Africa [7]. However, in lower and middle-income countries, such as Ethiopia, information on specific interventions, service utilization, and legislation is lacking [11]. In addition, there are only a small number of inaccessible rehabilitation facilities in the country. Besides, the lack of accessibility and employment opportunities are noted in almost all of the service areas [7], making it very challenging for people with disabilities to get out of the poverty-disability cycle. Despite the high burden, and interwoven challenges, little is known about disability in Ethiopia, particularly in the study area. This is believed to impose a great challenge for policy makers and planners to include people with disability in the inclusive development. Hence, the aim of this study was to assess the self-reported prevalence and factors associated with disability at the Dabat HDSS site.

## Methods

### Study design and setting

The study was conducted at the Dabat HDSS site where the census is conducted every 7 years in order to assess the changes in vital events, demography (living conditions, economic status, and health) of the population. The detailed activities of the HDSS site are mentioned elsewhere [12].This study is part of the re-census conducted from October to December 2014.

The Dabat HDSS site is located in Dabat District, northwest Ethiopia. The site was established in 1996. It covers a total of 13 kebeles, *smallest administrative unit in Ethiopia,* (9 rural and 4 urban) with 16,053 households and 67,395 inhabitants. The kebeles in the surveillance site were selected randomly, by taking all ecological zones (high land, middle land, and lowland) into account. Every household in the selected kebeles were targeted during the data collection period. Dabat HDSS is a full member of the International Network of Demographic Evaluation of Populations and Their Health (INDEPTH), a network of 44 HDSSs from the Global South.

### Study population and data collection

All permanent residents in the Dabat HDSS site were included in the study. The heads of each household were interviewed to collect the necessary information with regard to events that happened in the family. When a member of a family was found to have any form of disability, he or she was interviewed by trained, diploma graduate data collectors working in the research site using a structured and pretested questionnaire. The study utilized the re-census data.

Disability, the outcome variable, was defined according to the 2011 Labor Force Survey ad hoc module (LFS AHM) [13]. Hence, the status of disability is ascertained if a person has difficulty in carrying out any of the basic activities of hearing, seeing, walking, self-care, and cognition as parts of activities of daily living. A binary outcome of “yes” or “no” option was given to identify the presence or absence of disability. For example, a respondent would answer “yes” if he or she had difficulty in self-care, and “no” if there is no problem at all. Different independent variables (Table 1) were used to assess if there was an association with our outcome variable.

**Table 1:** Socio-demographic characteristics of the population of Dabat HDSS, northwest Ethiopia, October-December 2014 (*N* = 67,395)

Variables	Frequency	Percent
Sex		
Male Female	33,181 34,214	49.23 50.77
Age in years		
≤ 14 15 to 49 ≥ 50	28,956 29,807 8630	42.97 44.23 12.81
Residence		
Rural Urban	50,769 16,835	75.10 24.9
Marital status		
Under 10 years old Married Single Divorced Widowed Separated Cohabiting	20,089 21,814 19,746 2390 2369 917 68	29.81 32.37 29.30 3.55 3.52 1.36 0.10
Religion		
Orthodox Muslim Others^a^	64,940 2444 11	96.36 3.63 0.01
Ethnicity		
Amhara Tigre Others^b^	67,294 84 17	99.85 0.12 0.02
Education		
Not on education (<7 years) Unable to read and write Read and write Grade 1–4 Grade 5–6 Grade 7–8 Grade 9–10 Grade 11–12 Grade 12 and above	13,672 21,149 5541 10,960 4560 3590 4375 1957 1591	20.29 31.38 8.22 16.26 6.77 5.33 6.49 2.90 2.36
Doing work related physical activity		
Never Sometimes Most of the time	4498 16,414 16,872	12.00 43.00 45.00
Occupation		
No occupation(under 10 years) Students Farmers Employed permanent Private job Job seeker Merchant House maid Employed contract Retired Others(housewife, shepherd, disabled)	20,436 13,955 12,647 1951 1167 1056 656 623 328 296 33	38.45 26.26 23.80 3.67 2.20 1.99 1.23 1.17 0.62 0.56 0.05
Location		
Low land High land	22,380 45,015	33.2 66.8
Relation to the HH head		
HH head Housewife Son/daughter Sister/brother Mother/father Grandson/granddaughter Other relative Other non-relative	16,082 10,542 34,702 538 310 3095 1196 930	23.86 15.64 51.49 0.80 0.46 4.59 1.77 1.38
Family size		
1–4 5–9 10–15	24,512 41,667 1250	36.35 61.79 1.85
Wealth status		
Poorest quintile Second quintile Third quintile Fourth quintile Richest Quintile	9475 11,344 13,031 14,593 16,577	14.58 17.45 20.05 22.45 25.47

^a^ Catholic and Protestant

^b^ Oromo and Agaw

*HH* stands for Household

Food Security is defined as existing when “all people, at all times, have physical and economic access to sufficient, safe, and nutritious food to meet their dietary needs and food preferences for an active and healthy life”. In order to assess the Food security status of households, an 18 item community food insecurity accessible scale assessment tool was adapted from Household Food Insecurity Access Scale (HFIAS): Indicator Guide VERSION 3 and categorized into four levels using HFIS variables [14]. If the respondent answers “yes” to an occurrence question, a frequency of occurrence question was asked to determine whether the condition happened rarely (once or twice), sometimes (three to ten times) or often (more than ten times) in the past 4 weeks (food secure, mildly food insecure, moderately food insecure, severely food insecure). This scale has already been validated in Ethiopia [15].

The household wealth index was computed for urban and rural residents separately using the Principal Component Analysis (PCA). The urban wealth status was calculated by considering properties, like selecting household assets, while the only tropical livestock unit was used for the rural residents. The variables were initially screened using the commonality value. In the PCA, the Eigenvalue of greater than one, the KMO distribution, and in the final model, the common factor scores were summed and ranked in Poorest quintile, Second quintile, Third quintile, Fourth quintile, and Richest Quintile [16].

### Data processing and analysis

Data was entered into the Household Registration System (HRS) version 2.1 and analyzed using STATA version 14. Binary logistic regression was fitted to elicit factors associated with disability. The bivariable analysis was carried out, and variables with *p*-values of <0.2 were fitted to the multivariable logistic regression analysis. Both the crude odds ratio (COR) and the adjusted odds ratio (AOR) with the corresponding 95% Confidence Interval (CI) were used to show the strength of association. Finally, a p-value of <0.05 was used to declare statistical significance.

## Results

A population of 67,395 living in 16, 039 households were included in the study. About 34,214 (50.77%) respondents were female and 50,769 (75.1%) were rural dwellers. The mean age of the study subjects was 23.1 years (SD 19.1 years). Nearly half, 28,952 (42.96%), of the participants were under 15 years of age (Table 1).

**Fig. 1:**
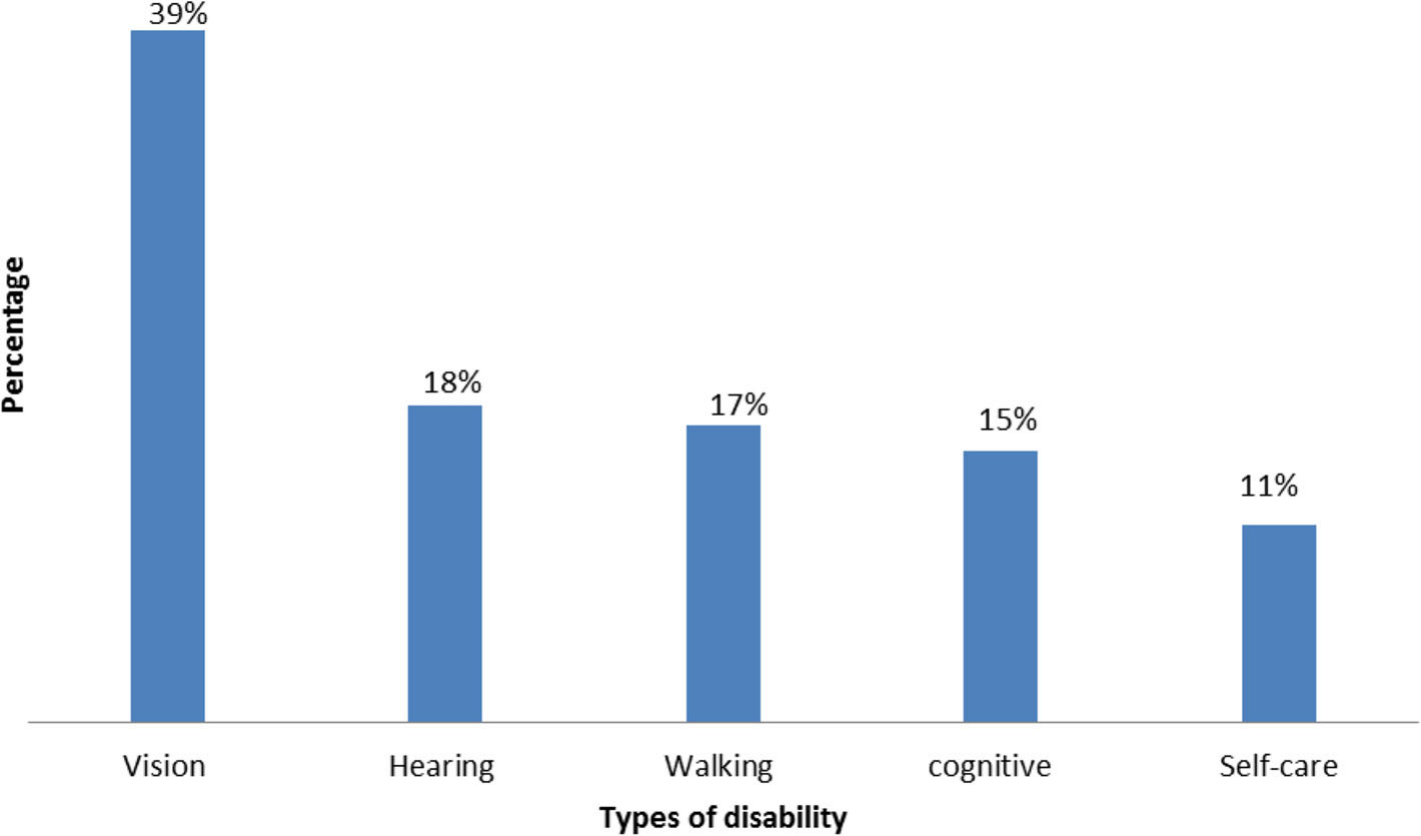
Number of individuals who reported disability among population of Dabat HDSS, northwest Ethiopia. October-December 2014 (*N* = 1228)

In this community, 1228 people were found with disabilities which corresponds to the overall prevalence rate of 1.82% [95%CI, 1.72, 1.92]. The mean (±SD) age of people with disabilities was 44.36 (±23.2) years. Regarding the types of disability, more than one-third, 534 (39%), were related to vision disability, followed by hearing 244 (18%) and walking 230 (17%) disabilities (Fig. 1). Moreover, cognitive and self-care disabilities account for 210 (15%) and 112 (11%), respectively, for the total disability. Among 1228 people who reported a disability, 11.5% of them have reported two or more types of disabilities.

Fall down injury and penetration by animal horn accounted for 155 (35.9%) and 88 (20.4%), respectively, of the common causes of disability (Table 2). Of the total study participants who experienced injury, 155 (35.9%) did not seek any treatment, while 85 (19.7%) went to traditional healers and 85 (19.7%) obtained some sort of treatment at home (Table 2). Regardless of gender, the proportion of vision disability increased with increasing age, while the rest of the disabilities were prevalent among the working age group (15–49 years old). One thousand three hundred sixty nine number of disabilities (20.3 cases per 1000 population) types of disabilities were reported among Dabat HDSS (Table 3).

**Table 2:** Causes of injury and post-injury health seeking behaviour among Dabat HDSS, North West Ethiopia, October-December 2014 (*N* = 432)

Variable	Frequency	Percentage
Causes of injury		
Fall	155	35.9
Burn	13	3.00
Poisoning	8	1.85
Drowning	1	0.23
Car accident	12	2.77
Sharp objects	57	13.12
Farming equipment	9	2.08
Hit by other person by stick	55	12.73
Animal Bite	34	7.87
Penetration by animals	88	20.4
Post injury health seeking behavior (N = 432)		
Did not need help	155	35.9
Treatment at home	85	19.6
Health post	17	3.9
Clinic	7	1.62
Health center	57	13.2
Hospital	26	6.02
Traditional Healer	85	19.7

**Table 3:** Types of disability by age and gender at Dabat HDSS, October-December 2014 (N = 67,395)

Types of disability (*N* = 1369)	Age < 5 years		Age 5–15 years		Age 15–49 years		Age ≥ 50 years		Total	
	M *N* = 4584	F *N* = 4503	M *N* = 9876	F *N* = 9985	M *N* = 14,601	F *N* = 15,206	M *N* = 4109	F *N* = 4521	M *N* = 33,181	F *N* = 34,214
	n (%)									
Cognitive *N* = 208	1 (0.02)	2 (0.04)	10 (0.10)	10 (0.10)	57 (0.39)	79 (0.52)	18 (0.44)	31(0.69)	86 (0.26)	122 (0.36)
Vision *N* = 534	3 (0.07)	0 (0.00)	16 (0.16)	21 (0.21)	56 (0.38)	94 (0.62)	143 (3.48)	201(4.45)	218 (0.66)	316 (0.92)
Hearing *N* = 244	2 (0.04)	0 (0.00)	15 (0.15)	13 (0.13)	52 (0.36)	67 (0.44)	41 (1.00)	54 (1.2)	110 (0.33)	134 (0.39)
Walking *N* = 230	3 (0.07)	4 (0.1)	20 (0.2)	6 (0.06)	51 (0.35)	50 (0.33)	51(1.24)	45 (1)	125 (0.38)	105 (0.31)
Self-care *N* = 153	3 (0.07)	4 (0.09)	11 (0.11)	9 (0.09)	30 (0.21)	33 (0.22)	36 (0.88)	27 (0.6)	80 (0.24)	73 (0.21)

The result of the multivariable logistic regression analysis showed that age, food security status, marital status, occupation, wealth status, and work-related physical activities were significantly and independently associated with disability. Consequently, the odds of getting disability were 4.49 times higher among elderly (≥ 50 years) population, compared to the younger ones (≤14 years) [AOR = 4.49; 95% CI: 1.95, 10.33]. The likelihood of disability was high among respondents with separated marital status [AOR = 7.52; 95% CI: 1.18, 47.84] and food in- secured households [AOR: 2.11; 95% CI: 1.59, 2.80]. However, being engaged in paid jobs was noted with lower odds of disability [AOR = 0.46; 95% CI: 0.28, 0.77] as compared to their counterparts. Similarly, respondents from a household with the highest wealth status [AOR: 0.76; 95% CI: 0.57, 1.00] and mostly engaged in work-related physical activities [AOR: 0.36; 95% CI: 0.27, 0.49] were found with lower odds of getting a disability (Table 4).

**Table 4:** Factors associated with disability among people at Dabat HDSS site, northwest Ethiopia, 2014

Variables	Disability yes n(%)	COR (95%CI)	AOR (95%CI)	Over all p-value
Sex				
Female	679 (1.98)		1	0.001
Male	547 (1.65)	0.82 (0.74,0.93)	0.98 (0.75, 1.28)	
Age in year				
≤ 14	137 (0.47)	1.00		<0.001
15 to 49	517 (1.73)	3.71 (3.03, 4.48)	1.57 (0.70, 3.53)	
> 50	572 (6.63)	14.9 (12.37, 18.1)	4.49 (1.95, 10.33)	
Wealth status				<0.001
Poorest quintile	314 (3.31)	1		
Second quintile	256 (2.26)	67.4 (0.56,0.79)	0.76 (0.57, 1.00)	
Third quintile	201 (1.54)	0.45 (0.38, 0.54)	0.75 (0.56, 0.99)	
Fourth quintile	238 (1.63)	0.48 (0.40, 0.57)	0.67 (0.51, 0.89)	
Richest quintile	195 (1.18)	0.34 (0.29, 0.42)	0.55 (0.41, 0.75)	
Residence				>0.05
Rural	909 (1.79)	1		
Urban	319 (1.89)	1.06 (0.93, 1.21)	0.99 (0.76, 1.30)	
Educational status				<0.001
Illiterate	44 (0.32)	1		
Can read & write	823 (3.89)	12.5 (9.25, 16.9)	0.85 (0.16, 4.51)	
Primary school	110 (1.99)	6 27 (4.41, 8.91)	0.6 (0.11, 3.19)	
High school	87 (0.79)	2.48 (1.72, 3.56)	0.6 (0.11, 3.18)	
Diploma and above	162 (1.01)	3.15 (2.26, 4.40)	0.3 (0.06, 1.63)	
Occupation				
Under age	114 (0.56)	1		
Student	120 (0.86)	1.54 (1.19, 1.99)	0.50 (0.29, 0.87)	<0.001
Farmer	277 (2.19)	3.99 (3.20, 4.97)	0.47 (0.30, 0.74)	<0.001
All type of paid job	81 (1.71)	3.10 (2.33, 4.14)	0.46 (0.28, 0.77)	<0.001
Unemployed	17 (1.61)	2.92 (1.74, 4.87)	0.66 (0.32, 1.33)	<0.001
Other	55 (16.72)	35 (25.39, 50.4)	1.25 (0.74, 2.11)	<0.001
Doing work related physical activity				<0.001
Never	400 (8.89)	1		
Sometimes	375 (2.28)	0.24 (0.21, 0.27)	0.59 (0.46, 0.77)	
Most of the time	275 (1.61)	0.17 (0.14, 0.19)	0.36 (0.27, 0.49)	
Food security				
Secure	427 (1.33)	1		
Mildly insecure	115 (1.58)	1.18 (0.96, 1.46)	1.18 (0.86, 1.62)	0.105
Moderately insecure	430 (2.26)	1.71 (1.49, 1.96)	1.49 (1.20, 1.86)	<0.01
Severely insecure	202 (3.52)	2.69 (2.28, 3.19)	2.11 (1.59, 2.80)	<0.01
Location of place				
High land	830 (1.84)	1		
Low land	396 (1.77)	0.96 (0.85, 1.08)	0.93 (0.75, 1.15)	0.520
Marital status				
Under age	79 (0.39)	1		
Married	460 (2.11)	5.45 (4.29, 6.93)	4.07 (0.67, 24.51)	<0.001
Single	312 (1.58)	4.07 (3.17, 5.21)	5.25 (0.87, 31.51)	<0.001
Divorced	143 (5.98)	16 (12.20, 21.3)	5.47 (0.90, 33.31)	<0.001
Widowed	205 (8.69)	23.9 (18.4, 31.2)	4.03 (0.66, 24.70)	<0.001
Separated	27 (2.74)	7 (4.58, 11.1)	7.52 (1.18, 47.84)	<0.001

## Discussion

This study is one of the largest studies conducted to document key health events in Ethiopia. The overall prevalence of disability was 1.82%. This burden corresponds to 7.6% of households reporting at least one person with disability.

Our finding is in line with the study done in Ghana, 1.8% [17]. This prevalence was lower than that of a previous study done in northern Ethiopia, which was 4.9% [18]. However, it was higher than the prevalence reported from other developing countries, such as Bahrain 0.4% [19] and Nepal 1% [20]. The observed discrepancy could be attributed to variations in the measurement of disability, methods utilized [21], and the primary goals of the surveys [22]. The burden of disability in our study corresponds to 7.6% of households reporting at least one person with disability. This figure is lower than what was reported by a national disability survey conducted in Zimbabwe, where 26% of households reported at least one member with disability [23].

In this study, vision disability accounts for 534 (39%) of the total disability burden. This finding is consistent with what was reported by other African countries: Nigeria 37% [9], South Africa, 32%) [24], and Zimbabwe (26%) [23]. This could be explained by poor eye hygiene, a level of access to health care, and health seeking behaviors in most developing countries, in Africa.

Out of the total reported disability, the proportion of hearing disability was 21%. This finding was comparable to that of the study done in South Africa, which was 20% [25], whereas it was 15% in Nigeria [9] and 12% in Zimbabwe [23]. The commonest causes of hearing disabilities in low and middle-income countries are infections from meningitis, measles, maternal rubella, febrile illnesses, and genetic traits [11]. In addition, another study claimed that increasing age was associated with hearing disability [26].

In our study, increasing age was significantly associated with disability. Similar to our finding, a previous study demonstrated that there was a strong association between older age and disability [22]. This is due to the presence of co-morbidities, chronic illnesses, and injuries. Similarly, a study in India indicates that co-morbidities, such as non-communicable diseases, increase with aging, which heightened the risk of developing disability [27]. A census of South Africa also showed that the prevalence of disability increased with age, the lowest (0.2%) was observed in the age group of 0–9 years, while the highest (27%) was among those aged 80 years and above [20, 24].

Household wealth status was inversely associated with disability. The result was supported by previous reports elsewhere [18, 25]. In fact, poor living conditions, unsafe working environments, poor nutrition, lack of access to clean water, basic sanitation, health care and education [28] are all linked to low socioeconomic status which further predisposes to the risk of developing a disability. A survey from 49 countries also indicated that disability was more prevalent in poorer than in the richest wealth quintiles [1]. Similarly, one of the local studies in Ethiopia showed that the prevalence of adult disability falls as wealth increases [1]. It was also reported that severe household food insecurity was associated with higher odds of getting a disability, which was supported by other findings [18, 26]. Access to nutrition for poor people is a serious problem in Ethiopia [29]. Evidence showed that access to good nutrition is directly related to food security, which has its own implications on the incidence of disability [22].

In this study, separated marital status increased the odds of having a disability. According to a study in the Netherlands on the population of 18,973 aged 15–74, married people were found with lesser odds of disability compared to their unmarried counterparts, single, divorced, or widowed [30]. Another study done in middle and low-income countries also showed that the prevalence was higher among divorced/separated/widowed adults than among the married/cohabiting respondents [2]. A disability doesn’t affect only individual health but also brings family/social crisis in a marriage. A previous study indicated that parents with a child on the Autistic Spectrum got divorced [31].

This is one of the biggest studies investigating the burden of disability in northwest Ethiopia and is believed to fill the knowledge gap and contribute to policy determination, clinical practice, and decision-making in the country. However, it is not free from some limitations. For instance, the study did not show the severity as well as the definitive causes of disability due to the cross-sectional nature of the study. In addition, the self-reported nature of this study means that the problems can be under or over reported. Disability is an umbrella term and the problems associated with it were not studied in depth. For example, the magnitude of limitations in each and every activity of daily living was not assessed.

## Conclusion

Even though the prevalence of disability in our finding is lower than the global statistics, the study reveals that there is a noticeable burden of disability at the Dabat HDSS site. Vision disability is the highest of all disabilities. Age, wealth status, food security status, marital, and occupational status were significantly associated with disability. Community education and creating a safe environment are key to prevent injuries which can result in disabilities. There is also a need to establish social protection strategies for the older, food in-secured, and poorest segments of the community. Furthermore, future researches need to cover a wider range and depth of disability to properly quantify disability and problems associated with it.

## Abbreviations

ADL: Activities of daily livings
AOR: Adjusted odds ratio
HDSS: Health and demographic surveillance system
HFIAS: Household Food Insecurity Access Scale
HRS: Household Registration System
INDEPTH: International Network of Demographic Evaluation of Populations and Their Health
LFS AHM: Labour Force Survey ad hoc module
PCA: Principal Component Analysis
SADPD: Secretariat for the African decade of disabled person
